# The Role of Peritoneal Macrophages in Endometriosis

**DOI:** 10.3390/ijms221910792

**Published:** 2021-10-06

**Authors:** Tamara N. Ramírez-Pavez, María Martínez-Esparza, Antonio J. Ruiz-Alcaraz, Pilar Marín-Sánchez, Francisco Machado-Linde, Pilar García-Peñarrubia

**Affiliations:** 1 Departamento de Bioquímica, Biología Molecular (B) e Inmunología, Facultad de Medicina, IMIB and Regional Campus of International Excellence “Campus Mare Nostrum”, Universidad de Murcia, 30100 Murcia, Spain; t.ramirezpavez@um.es (T.N.R.-P.); maria@um.es (M.M.-E.); ajruiz@um.es (A.J.R.-A.); 2 Servicio de Ginecología y Obstetricia, Hospital Clínico Universitario Virgen de la Arrixaca, IMIB, 30120 Murcia, Spain; Ibsyna@hotmail.com; 3 Servicio de Ginecología y Obstetricia, Hospital Clínico Universitario Reina Sofía, CARM, 30002 Murcia, Spain; fmachado@um.es

**Keywords:** endometriosis, peritoneal macrophages, inflammation

## Abstract

Endometriosis is an estrogen-dependent gynecological disorder, defined as the growth of endometrial stromal cells and glands at extrauterine sites. Endometriotic lesions are more frequently located into the abdominal cavity, although they can also be implanted in distant places. Among its etiological factors, the presence of immune dysregulation occupies a prominent place, pointing out the beneficial and harmful outcomes of macrophages in the pathogenesis of this disease. Macrophages are tissue-resident cells that connect innate and adaptive immunity, playing a key role in maintaining local homeostasis in healthy conditions and being critical in the development and sustainment of many inflammatory diseases. Macrophages accumulate in the peritoneal cavity of women with endometriosis, but their ability to clear migrated endometrial fragments seems to be inefficient. Hence, the characteristics of the peritoneal immune system in endometriosis must be further studied to facilitate the search for new diagnostic and therapeutic tools. In this review, we summarize recent relevant advances obtained in both mouse, as the main animal model used to study endometriosis, and human, focusing on peritoneal macrophages obtained from endometriotic patients and healthy donors, under the perspective of its future clinical translation to the role that these cells play on this pathology.

## 1. Introduction

Endometriosis is an estrogen-dependent gynecological disorder, defined as the growth of endometrial stromal cells and glands at extrauterine sites, mainly, but not exclusively, at the peritoneum and ovaries. Symptoms of endometriosis may vary depending on where the lesions are located, although chronic pelvic pain, dysmenorrhea and dyspareunia are the most common symptoms [1]. It is estimated that 10% of reproductive age women suffer endometriosis, with more than 200 million women affected worldwide [2]. In addition, this pathology seems to be closely related to the risk of infertility, mainly in women <35 years of age [3,4]. All this seriously affects the quality of life of patients with endometriosis [5] and entails annual costs of ~10,000€ per woman (Europe, USA and Australia), most of these expenses being due to the absence from work [6,7]. Furthermore, despite being a benign pathology, it has been demonstrated through observational studies that there is an association between endometriosis and some types of ovarian cancer [8,9]. Several studies suggest that the malignant transformation could be, in part, a product of the increased oxidative stress associated to the endometrial lesions [10]. This could be influenced by a decrease of M2 macrophages (Mϕ2) that express the antioxidant enzyme heme oxygenase (HO-1) [11]. In addition, it has been reported that iron could encompass the macrophages (Mϕ), and in this way, promote the malignant transformation of ovarian endometriosis [12].

Currently, endometriosis is considered a difficult to diagnose pathology, since its confirmation is only attained by invasive techniques such as laparoscopy. Regarding the available treatments, the most common are hormonal suppression or surgical excision/ablation of visible lesions, both of which are inefficient therapies due to the side effects that they entail and the frequent reappearance of endometriotic lesions, respectively [13].

**Figure 1:**
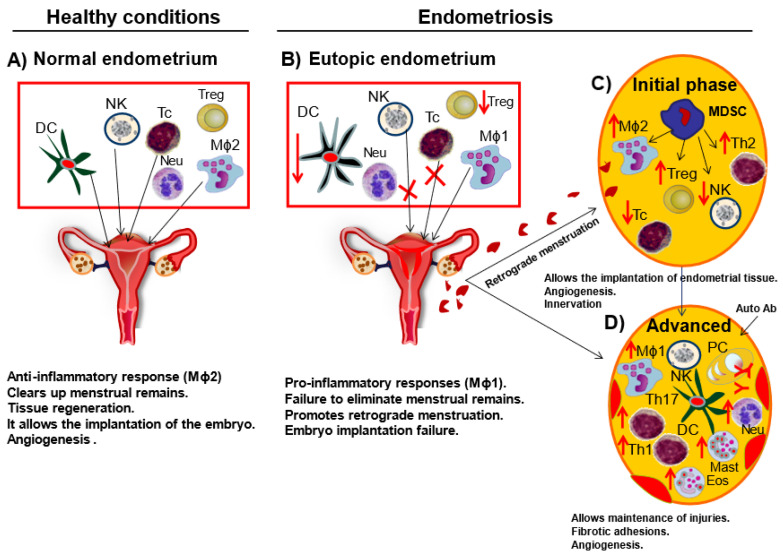
Immune system in endometriosis. (**A**) Under healthy conditions, Mϕ clear up cellular debris derived from menstruation and promote tissue regeneration, being anti-inflammatory Mϕ2 predominant in the normal endometrium. (**B**) In endometriosis, the main population seems to have a predominant Mϕ1 pro-inflammatory profile. The anti-inflammatory activity could be inhibited by a defective function of several cell types, including Treg, NK and cytotoxic T cells. This could allow the survival of endometrial cells, which can migrate to ectopic locations, developing in turn endometriotic lesions. (**C**) In the initial phase of endometriosis there is a higher Mϕ2 population and altered Treg, Th2 cells, and NK cells. This could allow the implantation and growth of endometrial cells. (**D**) Later, when endometriotic lesions are established, there could be an activation of several pro-inflammatory cell populations, such as Mast cells, Neutrophils, Eosinophils, Th1 and Th17. This inflammatory scenario would promote angiogenesis, fibrotic adhesions, and a failure of cells to clear the ectopic lesions. B cells, B lymphocytes; DC, dendritic cells; Eos, eosinophils; Mϕ, macrophages; Mast, mast cells; NK, natural killer cells; Neu, neutrophils; PC, plasmatic cells; T cells, T lymphocytes; T helper cells (Th1, Th2 and Th17); Treg cells, regulatory T cells.

Regarding the etiology of endometriosis, the most commonly accepted theory is that of Sampson, which suggests that during menstruation, the endometrial cells and tissue fragments reflow through the fallopian tubes and implant into the peritoneal cavity [14]. However, albeit retrograde menstruation is observed in most women [15], only 10% of them develop the pathology. In addition, endometriotic lesions have been widely observed in organs and tissues not accessible by retrograde menstruation in pre-menarchial girls [16]. This would indicate the existence of other mechanisms that favor the growth of ectopic endometrial tissue. In this regard, other theories have emerged that could explain this fact. The celomic metaplasia theory suggests that peritoneal celomic epithelial cells differentiate into cells similar to endometrium [17]. Whereas theory of stem cell origin proposes that the differentiation of mesenchymal stem cells within the menstrual backflow causes the development of endometriosis [16]. The observed ectopic endometrial tissue in female fetuses has given rise to the theory of embryogenic rests [18]. This theory suggests that defective embryogenesis processes would lead to the persistence of abnormal embryonic remnant of Müllerian ducts (from which the upper part of the vagina, uterus, and fallopian tubes develop) [19,20], which evolve into endometriotic lesions in response to estrogens. Finally, the theory of metastasis or vascular dissemination proposes that endometrial cells may enter the uterine vasculature or lymphatic system at menstruation and are transported to other sites [16]. In summary, the origin of this disease involves a series of individual factors of genetic [21,22] and environmental origin [23,24,25] along with a poor or abnormal immune response [19,26,27], which would allow the persistence and growth of endometrial tissue outside the uterus. In this regard, macrophages, as myeloid cells of the innate immune system, acting as key effectors in both innate and specific immune responses, seem to be key cells in the pathogenesis of this disease. These cells recognize and phagocytose pathogens, act as antigen presenting cells to activate T cells, and are involved in tissue repair among other varied functions [28]. It has been shown that macrophages (Mϕ) accumulate in the peritoneal cavity of women with endometriosis due to local secretion of chemotactic molecules [29], although while peritoneal macrophages are able to eliminate endometrial fragments under healthy conditions, this scavenger mechanism seems to be inefficient in endometriosis [30]. In this sense, the increased numbers of activated Mϕ and adaptive immune cells, T and B lymphocytes, together with the increase of inflammatory cytokines detected in patients with endometriosis [31], indicate the existence of a rather immunological/inflammatory dysregulation. In support to this theory, it has been reported that lymphocytes localized inside the ectopic tissue contribute to lesion growth, mostly associated with low ratios of Th1 to Th2 cells, depending on the stage of endometriosis [32], and a decreased natural killer (NK) cell cytotoxicity [33] (Figure 1). Furthermore, the increase in Killer Immunoglobulin-like Receptor (KIR) expression in peripheral blood NK cells may represent a risk factor for endometriosis [34].

For this reason, in order to find new diagnostic methods and treatments, the functional and phenotypic characteristics of the peritoneal immune system in endometriosis must be further studied.

## 2. Human Peritoneal Host Defenses

**Figure 2:**
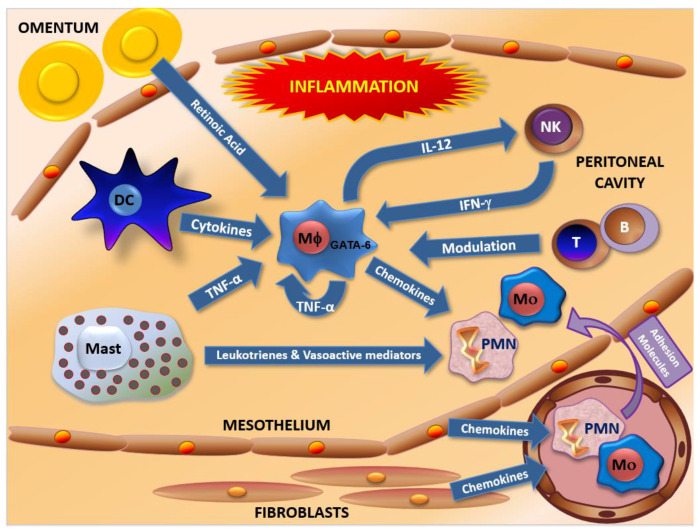
Crosstalk and interaction among cells involved in peritoneal host defenses. The defense of the abdominal cavity and the peritoneum depends on the leukocytes present in them, but also on mesothelial cells (MCs), fibroblasts and omentum. All these cells communicate through the secretion and membrane expression of several molecular mediators to coordinate the inflammatory process established to achieve host protection and the subsequent tissue repair. B, B cells; DC, dendritic cells; Mϕ, macrophages; Mast, mast cells; Mo, monocytes; PMN, polymorphonuclear cells; T, T cells.

The abdominal cavity is covered by the peritoneum, a smooth serosal membrane protecting this anatomical compartment against tissue injury and infections, reducing friction between bowel loops and other abdominal organs, and allowing diffusion of liquids, metabolites and immune cells [35]. The peritoneal layer is paved by a monolayer of epithelioid-like cells of mesenchymal origin, named mesothelial cells (MCs), which is the predominant cell type [36]. Below the layer of MCs there is an extensive network of capillaries and lymphatic vessels (Figure 2). Ultra-structural and functional studies have demonstrated that MCs not only secrete lubricant components such as surfactant and glycosaminoglycans to avoid friction and adherences between parietal and visceral surfaces, but also play an active role in peritoneal immune defenses [37]. Thus, in the presence of secreted TNF-α, IL-1β and IFN-γ by activated leukocytes, MCs produce a vast array of biologically active molecules including: lipid mediators (prostaglandins and prostacyclin), growth factors, cytokines, chemokines [38] and adhesion molecules [39,40] to recruit more leukocytes enabling leukocyte adherence and migration across the mesothelium (Figure 2). MCs also produce hyaluronan, which sequesters free radicals and initiates tissue repair [41] and other extracellular matrix molecules such as fibronectin, laminin, elastin, collagen and proteoglycans. MCs also regulate hemostasis in the presence of inflammatory stimuli, switching from a predominant fibrinolytic phenotype mediated by tissue plasminogen activator (tPA), urokinase PA (uPA) and uPA receptor (uPAR) toward a procoagulant state, by secretion of the inhibitor of the tissue plasminogen activator (PAI-1) and tissue factor [36,42]. Furthermore, it has been reported that MCs express MHC-II molecules and are able to phagocytose and present antigens to T lymphocytes [37,39].

The peritoneal wall also contains peritoneal fibroblasts that are located between peritoneal capillaries and mesothelial cells. In vitro studies have shown that peritoneal fibroblasts are able to produce inflammatory mediators such as prostaglandin E2 (PGE2), prostacyclin (PGI2), IL-6 and IL-8 in the presence of IL-1 and TNF-α [37]. Thus, these cells may also play a role in peritoneal host defenses [42] (Figure 2).

The peritoneal fatty tissue termed omentum connects the peritoneum with the spleen, stomach, pancreas, and colon. Murine omentum contains a high level of retinoic acid-converting enzymes that allow macrophages to be exposed to high amount of retinoic acid, a key factor to induce the expression of transcription factor GATA-6, specifically for the development of peritoneal macrophages in mice [43,44,45] (Figure 2).

Among all peritoneal cell types, leukocytes play a crucial role in the defense against microbial infections at this location. Studies on the physiology of human resident peritoneal leukocytes have been hindered by the difficulty of isolating these cells in healthy people, requiring aggressive surgical procedures. For this reason, most reported results on resident macrophages in homeostasis have been obtained from experimental animals, especially from the mouse model. Nevertheless, extrapolation from mice to human is not always feasible, especially in this particular field [46].

Studies on human peritoneal leukocytes in homeostasis are most usually performed from samples obtained from gynecological laparotomies or laparoscopies performed on healthy women [47,48] and patients with gallbladder stones without noticeable signs of inflammation [49], although the prevailing reported data have been obtained from continuous ambulatory peritoneal dialysis (CAPD) effluents [50,51,52].

Table 1 shows that among human peritoneal leukocytes, macrophages are the predominant cell type in healthy women and patients undergoing CAPD [47,49,50,51,52,53,54]. Recently, it has been reported that percentages of peritoneal macrophages decrease throughout childhood from 49.2% in neonates (>80% belonged to the CD14+ CD16^high^ subset) to 41.1% in adolescents (30% were CD14+ CD16^high^) [55]. The second, more numerous populations were constituted by T lymphocytes (predominantly T effector/memory cells, 45% CD45RO+). Moreover, the healthy human peritoneal compartment has a lower ratio of CD4+ to CD8+ T cells compared with that of peripheral blood lymphocytes, with an increased frequency of CD8+ T cells, and displaying a prevailing Th2 anti-inflammatory phenotype of CD4+ T cells [49,56]. Less information has been reported on the peritoneal content of NK and dendritic cells. Concretely, reported percentages of NK cells were 0.7–7.5% [47,49], and those for dendritic cells were ~9.3% [47]. Lower numbers have been also found for PMN cells (less than 5%) [47,49,50,51,52] and B lymphocytes (0.1–1%) [47,49]. It has been also shown that percentages of peritoneal B cells decrease throughout childhood from 0.7% (0–2 years) to 0.3% (10–18 years old) [55]. B-1 cells are innate-like B cells predominantly located in murine pleural and peritoneal cavity during fetal and neonatal development, display a wide specificity against ubiquitous bacterial antigens, and also recognize self-antigens and secrete IgM and IgA “natural” antibodies of low affinity. However, many ambiguities remain associated with the identification of this subset in humans [57]. 

Regarding resident mouse peritoneal macrophages, new important information on the ontogeny, phenotype, function, distinctive transcription factors and migratory activity have been reported [43,44,45]. Of note, it has been shown that tissue resident macrophages have a heterogeneous origin (from embryonic yolk sac and/or blood monocytes) as well as a different capacity for self-renewal. GATA-6, a recently described peritoneal macrophage-specific transcription factor, which is dependent on retinoic acid (highly produced by omentum located cells), induces the expression of specific genes, including programs for cell migration and adhesion, as well as cytokines secretion, such as TGF-β_2_ that, in turn, induces class-switching and IgA secretion by B-1 cells. Expression of GATA-6 in murine macrophages causes their peritoneal accumulation, self-renewal ability and expression of several peritoneal macrophage-specific genes such as *TGFB2* and *ASPA* in this location [43,44,45]. Hence, it seems necessary to assess whether the local tissue mediators and macrophage transcription factors that have been identified in mice play equal roles in the biology and development of human tissue-resident macrophages.

In recent years, our group has laid the foundations to isolate and determine the phenotypic and functional characteristics of peritoneal monocytes/macrophages (pMo/Mϕ) from healthy women [47]. This standardized procedure allows obtaining cells from a control group to be compared with the data obtained from women with endometriosis. Our results have confirmed the presence of three CD14/CD16 subpopulations of pMo/Mϕ in steady-state, which were previously detected in ascites fluid of cirrhotic patients [58]. Among them, the more complex CD14^high^CD16^high^ subset displayed the highest number of GATA-6-positive cells, a higher phagocytic/oxidative activity and expression of membrane receptors involved in phagocytosis, antigen presentation, activation, and co-stimulation. The intermediate subpopulation expressed an intermediate level of those markers, while the classical-like subset was more alike to the corresponding subset of peripheral blood monocytes. Finally, a similar level of pro-inflammatory Mϕ1 and anti-inflammatory Mϕ2 polarization markers was detected, which is consistent with a basal pre-activated state to quickly respond against any aggression to preserve peritoneal homeostasis.

## 3. Macrophage Phenotype in Endometriosis

Macrophages are very versatile cells not only in terms of their functions, but their phenotype has also been shown to vary depending on the type of immune response required against different types of tissue injuries. Thus, two main phenotypes to which macrophages can polarize have been broadly described, namely, the classical phenotype or M1 (CD40+/CD80+/CD86+/HLADR+) and the alternative phenotype or M2 (CD163+/CD206+/CD204+) [59]. Mϕ1 induced by interferon gamma (IFN-γ), tumor necrosis factor α (TNF-α) or bacterial lipopolysaccharide (LPS) [60] secrete proinflammatory cytokines and chemokines that promote the initial response against infections, triggering the inflammatory response and the proliferation of myoblasts [61]. In contrast, Mϕ2 display an anti-inflammatory and tissue repair character, mainly promoted by IL-4, IL-10, IL-13, or transforming growth factor-β (TGF-β) [60] and once activated, they secrete anti-inflammatory cytokines, growth factors, and other repair components [61]. Based on this, Mϕ2 are considered immunosuppressive cells that promote tissue repair, angiogenesis and tumor growth [60].

Most of the knowledge on the role played by the immune system in human inflammatory diseases has been obtained from the peripheral blood compartment. However, studying macrophages in an inflammatory scenario is pivotal to a better comprehension of the physiopathology of those diseases. Hence, to understand the role of macrophages in the development of endometriosis, it is important to elucidate their behavior in the endometriotic environment and determine whether there is any functional or phenotypic modification that may explain their failure, in order to prevent the development of ectopic lesions.

Recent findings have indicated that macrophages polarized to the M2 tissue repair phenotype are predominant in the peritoneal environment of women with endometriosis [62,63] (Figure 1). Some studies suggest that this polarization could be influenced by IL-33, which is increased in endometrial lesions compared to the endometrium of healthy women [64,65]. However, it should be noted that, as in vivo resident macrophages present a wide spectrum of tissue and/or disease-specific phenotypes, the classification of M1/M2 macrophages may be over simplified, and thus researchers typically refer to this as “a phenotypic trend” of these populations [66]. Data on the phenotypic profile of macrophages present in the peritoneal cavity of women with endometriosis (Table 2) are mainly based on those markers that are altered in comparison with the corresponding levels of healthy women [30,59,67,68,69,70].

Nie et al. [59] reported a decrease in the percentage of Mϕ1 (CD86+) and an increase in the ratio of CD163+/CD86+, Mϕ2 in the peritoneal lavage of patients with endometriosis, especially in those with advanced disease, suggesting that the presence of retrograde menstruation into the abdominal cavity could induce an immune tolerance state of pMϕ [71]. Furthermore, an increase in CD14^high^ macrophages [67] and CD68 expression [68] has been observed in the peritoneal fluid of women with endometriosis. Recently, Hudson et al. [72] have also reported that pMo/Mϕ are composed of two populations of cells that exhibit major differences in the levels of the CD14 and CD68 markers, classified as CD14^low^/CD68^low^ and CD14^high^/CD68^high^ subsets. However, only the CD14^low^/CD68^low^ subpopulation undergoes changes during the development of endometriosis, showing an increased Mϕ2 type response.

Analysis of others phenotype markers as HLA-DR (active Mϕ), NLC-MACRO and HAM56 (later Mϕ differentiation) have shown higher expression in pMϕ from women with endometriosis [68,69].

The expression of Fas antigen (CD95), involved in apoptosis-mediated death, has been also shown to be increased in pMϕ in endometriosis [69]. As Fas antigen is a surface protein with proapoptotic properties, an increase in its expression could trigger an imbalance between apoptosis and activation/surveillance/proliferation of immune and endometrial cells [69].

The existence of an aberrant expression of surface proteins involved in phagocytic response by pMϕ of women with endometriosis compared with healthy women has been reported. Thus, the Signal Regulatory Protein-α (SIRP-α), a phagocytosis inhibitory receptor, and the CD200 receptor (CD200R), which together with its ligand CD200 form an immunosuppressive complex, are significantly overexpressed in pMϕ of women with endometriosis [30,70]. In addition, CD200 has been linked to ectopic endometrial deposits [73]. On the other hand, CD36, a scavenger receptor involved in multiple physiological processes, including the clearance of oxidized low-density lipoprotein and apoptotic neutrophils, has significant lower expression in pMϕ of women with endometriosis [30]. The aberrant expression of these surface proteins associated to endometriosis seems to trigger the decrease of pMϕ phagocytic capacity [30,70].

The cytoplasmic inducible nitric oxide synthase (iNOS) is the predominant form of NOS in Mo and Mϕ and is responsible for nitric oxide (NO) production by the enzymatic conversion of L-arginine to L-citrulline [74]. It has been shown that pMϕ overexpress iNOS in endometriosis [75]. Even though iNOS is not a surface marker, its overexpression is important since it is known that high levels of NO can negatively influence fertility [76]. Therefore, the increase in the iNOS expression can trigger an increase in the NO levels and consequently affect the fertility of women with endometriosis.

Thus, knowing the expression of cell surface markers and the cytokine secretion profile of pMϕ in endometriosis is crucial to better understand this pathology.

## 4. Phenotype of Macrophages in Animal Models of Endometriosis

Several studies performed in animal models have analyzed the phenotype of pMϕ in endometriosis. In support of reported results from women with endometriosis, in a murine model it has been described how exosomes from ectopic stromal cells of female mice with endometriosis lead to the polarization of pMϕ towards a phenotype similar to M2, presenting a decreased phagocytic capacity [77].

Contrarily to the phenotype found in pMϕ, some investigations have suggested that macrophages located in the eutopic endometrium of women with endometriosis would be biased toward the M1 phenotype compared to the endometrium of healthy women [78,79] (Figure 1). However, in contrast to these findings, in a model of endometriosis in Rhesus Macaques, Smith et al. [80] found that macrophages from both eutopic and ectopic endometrium tended to be polarized to the M2 phenotype compared to the corresponding macrophages obtained from healthy macaques. On the other hand, in a murine model of endometriosis, Johan et al. [81] showed that the transference of human-recruited-macrophages-to-endometrial-tissue to animals changed from a predominant M1 phenotype at the beginning of the lesion, to a predominant M2 phenotype, expressing arginase 1 on day 7, and the CD204 scavenger receptor on day 14 of lesions development as depicted in Figure 1.

Altogether, these findings suggest the existence of different Mϕ populations in eutopic and ectopic endometrial tissue in endometriosis. In addition, these populations could undergo phenotypic changes along the progression of the disease. Besides, there seems to be a phenotypic difference between resident pMϕ and monocyte-derived macrophages. In this regard, Yuan et al. [82] based on the expression of nitric oxide synthase (NOS2) and CD206 in a murine model of endometriosis, reported that resident pMϕ exhibited a pro-inflammatory activation state, whereas monocyte-derived macrophages had a restorative nature. More recently, Hogg et al. [83] have shown that macrophages located in endometriotic lesions come from endometrial tissue lining the uterine cavity, pMϕ, and blood monocytes derived from bone marrow progenitors. Endometriosis induces a regular recruitment of Mo that differentiate into Mϕ, which are different from those that are usually present in the peritoneal cavity. Depletion of different populations revealed that endometrial Mϕ are “pro-endometriosis” cells, whereas monocyte-derived peritoneal macrophages are “anti-endometriosis”, protecting the abdominal cavity from the establishment of the injury. Ono et al. [84] have reported that depletion of CD206+ macrophages in a mouse model, significantly decreased the formation of endometriotic-like lesions. Their data suggested that CD206 Mϕ enhance the development of endometriotic-like lesions by inducing angiogenesis through secretion of VEGFA and TGF-β1.

## 5. Role of Cytokines Secreted by Macrophages in Endometriosis

As described above, cumulative findings have led endometriosis to be considered as an inflammatory pathology, closely related to an abnormal function of macrophages. For this reason, studying the profile of cytokines secretion by these cells has become a key objective. In this regard, various groups have attempted to elucidate the role of some of these cytokines in the development and maintenance of endometriotic lesions. Table 3 provides the characteristic altered pattern of cytokines secretion from pMϕ of women with endometriosis compared to those of healthy women [85,86,87,88,89,90,91,92,93,94,95]. The increased level of several cytokines in the peritoneal cavity could support the presence of a dysregulated state of Mϕ and other immune cells, contributing to the maintenance of a chronic inflammatory environment.

IL-6 is the most studied pro-inflammatory cytokine in endometriosis [85,86,87,88,89]. IL-6 is related to: monocyte recruitment [96], in vitro polarization of pMϕ to the M2 phenotype [59], the number of lesions generated in a murine model of endometriosis [96], and together with its receptor (IL-6R), has been implicated in endometrial stromal cells (ESC) growth regulatory signaling in vitro [97].

On the other hand, it has been reported that there is an increase in the levels of the anti-inflammatory cytokine IL-10 in the peritoneal cavity of patients with endometriosis [88,91]. In support of these findings, it has been shown that depletion of IL-10 in a surgically induced endometriosis murine model, led to a significant decrease of the endometrial lesions size, while the IL-10 administration promoted the growth of endometrial lesions in this model [98]. These results suggest that IL-10 can suppress the immune response against endometrial implants, contributing to the development of endometriosis; which could be the consequence of promoting angiogenesis or suppressing anti-self-responses in endometrial lesions [98]. However, Jeljeli et al. [99] have shown that LPS^low^-memory macrophages display an anti-inflammatory profile, alleviate endometriosis growth in mice, and dampen fibro-inflammatory properties of human endometriotic cells in an IL-10-dependent manner.

The levels of IL-8 and MCP-1 are also increased in the peritoneal cavity of women with endometriosis [85,93]. Both cytokines have been involved in the recruitment of Mϕ in endometriosis and other pathologies [100].

The role of IL-1β and IL-2 in the endometriosis development is still unclear. Both correspond to pro-inflammatory cytokines that are increased in the peritoneal fluid of patients with endometriosis [88,94,95].

Tumor necrosis factor α (TNF-α), a key factor in the inflammatory immune response, is involved in the endometriosis development. It has been reported that TNF-α is present in the peritoneal cavity of patients with endometriosis, and its levels are significantly higher in the early stages of the pathology [93]. Furthermore, it has been reported that TNF-α increased endometrial stromal cell adhesion to the extracellular matrix components [101].

Transforming growth factor β (TGF-β) is generically involved in immunosuppressive activities [102]. It is also increased in the peritoneal cavity of patients with endometriosis [88,90,93], and has been related to the pathogenesis of endometriosis. Hull et al. [103] described that TGF-β could regulate the size of endometriotic lesions through its effect on the phenotype and function of Mϕ. Hence, a murine model with TGF-β deficiency showed a decrease in the size of the ectopic lesions with significantly fewer Mϕ in them compared to mice with normal TGF-β expression levels. Moreover, increasing levels of TGF-β in the peritoneal cavity of both rats and women with endometriosis have been reported to be associated with increased survival, binding, invasion, and proliferation of ectopic endometrial cells during the development of endometrial lesions [104,105]. Furthermore, Yang et al. [106] reported that the interaction between ESC and Mϕ could decrease the cytotoxicity of natural killer (NK) cells, possibly stimulating the secretion of IL-10 and TGF-β by Mϕ, and in this way, they could favor the immunological escape of ectopic fragments and promote the appearance and development of endometriosis.

On the other hand, it has been reported that the serum levels of IL-8 [85,93], MCP-1 [93], IL-10 [88,91,95], IL-1β [88] and TGF-β [88,93] are also increased in patients with endometriosis. Nevertheless, contradictory results for levels of IL-10 [88,89,90,91], IL-1β [86,88,89], IL-2 [91,95] and TNF-α [85,90,93] in the peritoneal fluid of women with endometriosis has been also reported. Those differences could be mainly due to the control groups used, since none of those studies use a control group of total healthy women. The reported control groups are often heterogeneous, including a mix of tubal ligations (healthy women), but also women with unexplained infertility, myomas and hysterectomies due to uterine bleeding. Moreover, during laparoscopy the presence of peripheral blood contamination could be a common issue.

Recently, Voltolini Velho et al. [92] have analyzed the content of several cytokines in the peritoneal fluid of women with endometriosis. Their results showed that IL-1β, IL-1RN, IL-2, IL-4, IL-8, IL-10, IL-12 (p70), IL-17α, FGF2, G-CSF, MCP-1, MIP-1α, TNF-α and neopterin were significantly increased compared with controls. They also found a correlation between IL-2, MCP-1, MIP-1α, TNF-α and the severity of endometriosis. Their data showed an increase of T_H_1–T_H_2 ratio caused by the increased amount of TNF-α and IL-2 in women with severe endometriosis.

## 6. Molecules Involved in the Recruitment of Peritoneal Macrophages in Endometriosis

The contribution of various molecules in the Mϕ recruitment to the peritoneal compartment has also been studied. As described above (Table 3), some studies have reported that MCP-1 chemokine levels are increased in the peritoneal cavity of women with endometriosis [85,92,93]. MCP-1 seems to contribute to the paracrine and autocrine activation of macrophages, triggering their accumulation into the peritoneal cavity of patients with the disease [93]. Furthermore, other studies have described that endometriosis-induced expression of MCP-1 increases the recruitment of Mϕ in humans and rats [107,108]. Recently, Mei et al. [109] reported from primary cultures that the secretion of MCP-1 and IL-22 by ectopic ESC stimulated by natural killer (NK) cells contributed to the recruitment of macrophages. In addition, Cakmak et al. [110] demonstrated that MCP-1 in conjunction with IL-8, which is involved in Mϕ recruitment in cancer, are involved in the onset and development of endometriosis through the p38 MAPK signaling pathway.

RANTES (CCL5), another member of the CC chemokine family, could also be involved in the recruitment of macrophages towards endometrial lesions. Thus, Wang et al. [111] demonstrated that its increase produced by cells associated with the endometriotic focus could recruit macrophages into the ectopic environment and also induce their immune tolerance.

On the other hand, hypoxia-induced Semaphorin 3A (Sema3A), a local secreted protein with axono-repulsive action, has also been involved in Mϕ recruitment in endometriosis. It has been shown that Sema3A is present in endometrial lesions and may contribute to the regulation of aberrant sympathetic innervation in peritoneal endometriosis [112]. In addition, another study showed that Sema3A can recruit Mϕ through the Sema3A/Nrp-1 (Neuropilin-1) signaling pathway, guiding them to a hypoxic environment like endometriosis lesions [113].

Regulatory T cells induce polarization of pro-repair Mϕ by secreting soluble fibrinogen-like protein 2 (sFGL2) into the endometriotic milieu. sFGL2-induced pro-repair Mϕ promote Th2 and Tregs differentiation, creating a positive feedback [114].

## 7. Phagocytosis Defects of Macrophages in Endometriosis

Phagocytosis is one of the main antimicrobial defense mechanisms of the innate immune system, mainly exerted by neutrophils, monocytes, and macrophages. This mechanism allows the elimination of both pathogens and cellular debris and, therefore, under normal conditions, pMϕ should be able to eliminate ectopic endometrial tissues migrated there by retrograde menstruation. However, since Mϕ are not capable of eliminating the ectopic endometrium in endometriosis, it is thought that there is a defect in their phagocytic ability.

Xie et al. [30] from a study carried out in human pMϕ and peripheral blood mononuclear cells, concluded that the eutopic endometrium could reduce the phagocytic capacity of pMϕ in women with endometriosis by increasing the expression of the Signal Regulatory Protein-α (SIRP-α), and decreasing the corresponding expression of the CD36 scavenger receptor (Table 2). In support of the role played by SIRP-α in modulating phagocytosis, recent studies have described that the binding of SIRP-α to its receptor CD47 regulates neural networks, immune homeostasis, tumor development and induce impairment of the phagocytic capacity of alveolar macrophages after the resolution of primary bacterial or viral pneumonia, as it initiates a cascade of events leading to macrophage inhibition of phagocytosis [115,116]. Therefore, up-regulation of SIRP-α/CD47 signaling may allow aberrant cells to escape immune surveillance [30]. CD47 also acts as a receptor for Thrombospondin 1 (TSP-1), a molecule with antiangiogenic activity, which has also been implicated in the decrease of the phagocytic capacity of pMϕ in primary cultures [117].

Regarding CD36, there is multiple evidence of its role in modulating phagocytosis in various diseases [118,119]. Hence, it has been described that there is a lower expression of CD36 in pMϕ of women with endometriosis [30] (Table 2), producing a lower efficiency to eliminate apoptotic cells compared to that of Mϕ from healthy women [120]. Furthermore, CD36 blockade impairs the phagocytic ability of normal Mϕ; and its overexpression restores the phagocytic capacity of Mϕ from women with endometriosis [120]. It has also been described in a murine model, that treatment with prostaglandin E2 (PGE2) inhibited CD36-dependent phagocytosis in pMϕ and exacerbated endometriotic lesions [121]. PGE2 also regulated matrix metalloproteinase 9 (MMP-9), an enzyme involved in the elimination of cellular debris, which has a low expression in pMϕ of women with endometriosis [122]. TGF-β could also modulate the expression of CD36, since inhibition of TGF-β in THP-1 cell-derived macrophages treated with a eutopic endometrial homogenate, improved the expression of CD36, therefore TGF-β could be a potential therapeutic target for the treatment of endometriosis [30].

On the other hand, as shown in Table 2 the expression of CD200 receptor (CD200R) is increased in pMϕ of women with endometriosis, and there is also an increase in the expression of CD200 (CD200R ligand) in ectopic endometrial tissues. Under healthy conditions, the expression of CD200 is induced by estrogens. Furthermore, in vitro cultures of differentiated THP-1 cells have shown that increased levels of CD200 decrease the phagocytic capacity of Mϕ and suggest that the CD200/CD200R complex exerts an immunosuppressive function [70].

## 8. Dependence of Estrogens and Their Receptors in the Development of Endometriosis

Estrogens are key mediators of endometrial cell homeostasis since they regulate both the eutopic and ectopic endometrium. For this reason, any estrogenic deregulation can lead to endometrial pathologies. The endometrial and other target cells can recognize estrogens by expressing estrogen receptors (ER), which are classically divided into alpha (ERα) and beta (ERβ) receptors, both acting as nuclear receptors [123]. Regarding the function of these receptors, it is known that ERα is the main responsible for the modulation of genes related to cell growth, while ERβ plays an important role in the progression of the cell cycle and apoptosis [124].

Endometrial lesions aberrantly express several steroidogenic enzymes, including aromatase and 17β-hydroxysteroid dehydrogenase (17β-HSD), resulting in increased synthesis and decreased metabolism of 17β-estradiol [125] leading to high 17β-estradiol levels in endometrial lesions [126]. This, in conjunction with the increased expression of ERβ [127] in both ovarian and peritoneal lesions, could over-activate estrogen-dependent signaling pathways in endometrial tissues, stimulating its corresponding biological effects. Furthermore, ERβ has been shown to be necessary for the progression of endometriosis in mice [128]. Furthermore, it has been reported that there is a high expression of both ERα and ERβ in pMϕ of women with endometriosis compared to healthy women [68]. The same study suggests that ERβ would play a role in the basal production of proinflammatory cytokines, while ERα would function as an inducer of the inflammatory response associated with endometriosis [68].

Regarding the mechanism by which ERβ contributes to the development of endometriosis, it has been described that ERβ modulates the production of MCP-1 by ESCs, through signaling pathways mediated by nuclear transcription factor κB (NF-κB), and would therefore recruit macrophages in ectopic lesions to promote endometrial pathogenesis [62]. Furthermore, it has been shown that most proteins that interact with ERβ are involved in inflammation and apoptosis signaling, and that an increased expression of ERβ by endometrial Mϕ contributes to the pathogenesis of the endometriosis by regulating the mechanisms involved in both apoptosis and activation of inflammasomes [129]. Additionally, it has been observed that estrogen-ER interaction can regulate the production of IL-6 and TNF-α of lipopolysaccharide (LPS) activated pMϕ from patients with endometriosis [130].

It has also been described that 17β-estradiol could stimulate the secretion of colony stimulating factor 1 (CSF-1) and MCP-1 by peripheral nerves, thus attracting Mϕ towards endometriotic lesions [131].

## 9. Inflammation, Innervation, and Associated Pain in Endometriosis

Pelvic acute chronic pain is the more frequent symptom associated with endometriosis, possibly caused by the inflammatory process and the high innervation present in endometrial lesions [132]. In addition, it has been shown that the presence of nerve fibers associated with endometriosis would be related to the severity of dysmenorrhea [133].

High innervation also appears to be closely related to Mϕ recruitment, since a higher number of inflammatory cells near nerve fibers has been observed in women with endometriosis [134]. In addition, it has been shown that Mϕ recruitment to nerve fibers within endometriotic lesions facilitates the development of inflammatory pain [134]. This suggests a direct attraction of macrophages that could be mediated at least in part by colony-stimulating factor 1 (CSF-1) and MCP-1 secreted by nerve fibers, which have been shown to increase macrophage migration in a murine model [131]. Notably, it has been recently reported in women with endometriosis that the CD14^high^ subset of pMϕ is negatively correlated with pain scores, while the CD14^low^ subpopulation is positively correlated with pain scores [67].

On the other hand, miR-146b (miRNA) has been shown to be significantly increased in samples of peritoneal fluid and blood serum from patients with this pathology. Furthermore, this miRNA has been associated with the risk of pain [135,136], suggesting a possible role of miR-146b in the etiology of endometrial pain. Other molecular analysis revealed that this effect could be produced by the inhibition of M1 macrophage polarization mediated by the transcriptional factor IRF5 (necessary for polarization to Mϕ1) [135].

## 10. Conclusions and Perspectives

Endometriotic lesions are characterized by an abnormal inflammatory environment and chronic endometrial cell growth. Given that most ectopic endometriosis lesions develop in the peritoneal cavity, this particular immunological context should play the most relevant role in this disease. In this sense, pMϕs are critically involved in the development of endometriotic lesions since they are the predominant immune cells within the peritoneal fluid. The study of Mϕs located in the inflammatory setting is essential to achieve a better understanding of the pathophysiology of endometriosis. Therefore, to understand the role of Mϕs in the development of endometriosis, it is important to elucidate their behavior in endometriotic lesions and determine the functional and phenotypic modifications that may explain their failure, in order to prevent the development of ectopic lesions. Taken together, most of the reported findings suggest the existence of different populations of Mϕ in eutopic and ectopic endometrial tissue. Endometriosis leads to polarization of pMϕ towards a phenotype similar to M2 in the initial phase and M1 in advanced stages, presenting a decreased phagocytic capacity. Furthermore, there appear to be phenotypic differences between resident pMϕ and monocyte-derived Mϕs. Increased levels of various cytokines in the peritoneal cavity could support the presence of a dysregulated state of pMϕ and other immune cells, contributing to the maintenance of a chronic inflammatory environment.

Macrophages are potential diagnostic and therapeutic targets due to their pathogenic role in a large number of pathologies, including endometriosis. Therefore, the phenotypic, functional and regulatory identification of Mϕs that promote endometriosis is essential to achieve the development of diagnostic and therapeutic procedures specifically directed at Mϕs associated with this disease.

## Figures and Tables

**Table 1:** Percentages of human peritoneal leukocytes.

Cell type	Monocytes/Mϕ (%)	Lymphocytes (%)				NKs (%)	DCs (%)	PMNs (%)	Reference
Subjects		Total	T Cells	CD4/CD8	B Cells				
**Healthy Controls (Women)**	89.0 ± 2.0	8.4 ± 1.6	—	—	—	—	—	—	[53]
	90.1 ± 1.9	—	—	—	—	—	—	—	[54]
	93.2 ^1^	—	—	—	—	—	—	n.d.	[50]
	85.0–95.0 ^2^	—	—	—	—	—	—	n.d.	[52]
	53.0 ± 26.5	—	23.5 ± 27.4	—	0.1 ± 0.2	0.7 ± 0.9	9.3 ± 11.0	2.5 ± 5.1	[47]
**Healthy Controls (Gallbladder)**	21.0–61.0 ^2^	—	37.0–49.0 ^2^	0.48	0.0–2.0 ^2^	0.0–18.0 ^2^	—	<5.0	[49]
**CAPD Effluent Patients**	86.5 ^1^	—	—	—	—	—	—	3.4 ^1^	[50]
	84.0 ^1^	10.0 ^1^	—	—	—	—	—	4.0 ^1^	[51]
	>60.0	—	—	—	—	—	—	—	[52]
**Healthy Controls (Infants)**	49.2 ± 7.1	22.5 ± 5.4	14.5 ± 4.5	—	0.7 ± 0.5	—	—	—	[55]
**Healthy Controls (Children)**	42.9 ± 11.1	33.1 ± 9.6	26.6 ± 9.8	—	0.8 ± 0.7	—	—	—	[55]
**Healthy Controls (Adolescents)**	41.1 ± 8.7	36.6 ± 7.1	31.5 ± 7.6	—	0.3 ± 0.1	—	—	—	[55]

Data represent ratios (%) of different cell types as Mean ± SEM, approximate value ^1^ or ranges ^2^; n.d. = not detectable.

**Table 2:** Surface markers found in peritoneal macrophages from women with endometriosis.

Marker	Endometriosis vs. Health Comparison	Statistical Significance (*p* value)	Technique Used	Reference
CD86	Diminished	**	Flow Cytometry	[59]
Ratio CD163+/CD86+	Increased	**		[59]
CD14^high^	Increased	*		[67]
CD68	Increased	***	Immunohistochemistry	[68]
HLA-DR	Without changes	n.s.	Flow Cytometry	[59]
	Increased	**		[69]
NLC-MACRO	Increased	***	Immunohistochemistry	[68]
HAM56	Increased	***		[68]
CD95 (Fas)	Increased	*	Flow Cytometry	[69]
SIRP-α	Increased	**	Immunoblot	[30]
CD200R	Increased	*		[70]
CD36	Diminished	**		[30]

* = *p* < 0.05; ** = *p* < 0.01; *** = *p* < 0.001; n.s. = no significant difference.

**Table 3:** Variation of cytokines present in the intraperitoneal space of women with endometriosis vs. healthy women.

Cytokine	Comparison	Endometriosis vs. Health Significance	Reference
IL-6	Increased	*	[85,86,87]
		***	[88,89]
IL-10	Without changes	n.s.	[89,90]
	Increased	***	[88]
		*	[91,92]
IL-8	Increased	**	[85,92]
		***	[93]
MCP-1	Increased	*	[85,92]
		***	[93]
IL-1β	Without changes	n.s.	[86,90]
	Increased	**	[92]
		***	[88,94]
IL-2	Without changes	n.s.	[91]
	Increased	*	[95]
TNF-α	Without changes	n.s.	[85,90]
	Increased	*	[92,93]
TGF-β	Increased	***	[88,93]
		*	[90]

* = *p* < 0.05; ** = *p* < 0.01; *** = *p* < 0.001; n.s. = no significant difference.

## Data Availability

Not applicable.
